# Supplementary figures and images for: Interferon-Inducible Guanylate-Binding Protein 5 Inhibits Replication of Multiple Viruses by Binding to the Oligosaccharyltransferase Complex and Inhibiting Glycoprotein Maturation
Source: bioRxiv. 2024 May 3:2024.05.01.591800. Preprint. [Version 1] doi: 10.1101/2024.05.01.591800 (PMC11092618; doi:10.1101/2024.05.01.591800)

Figure S1

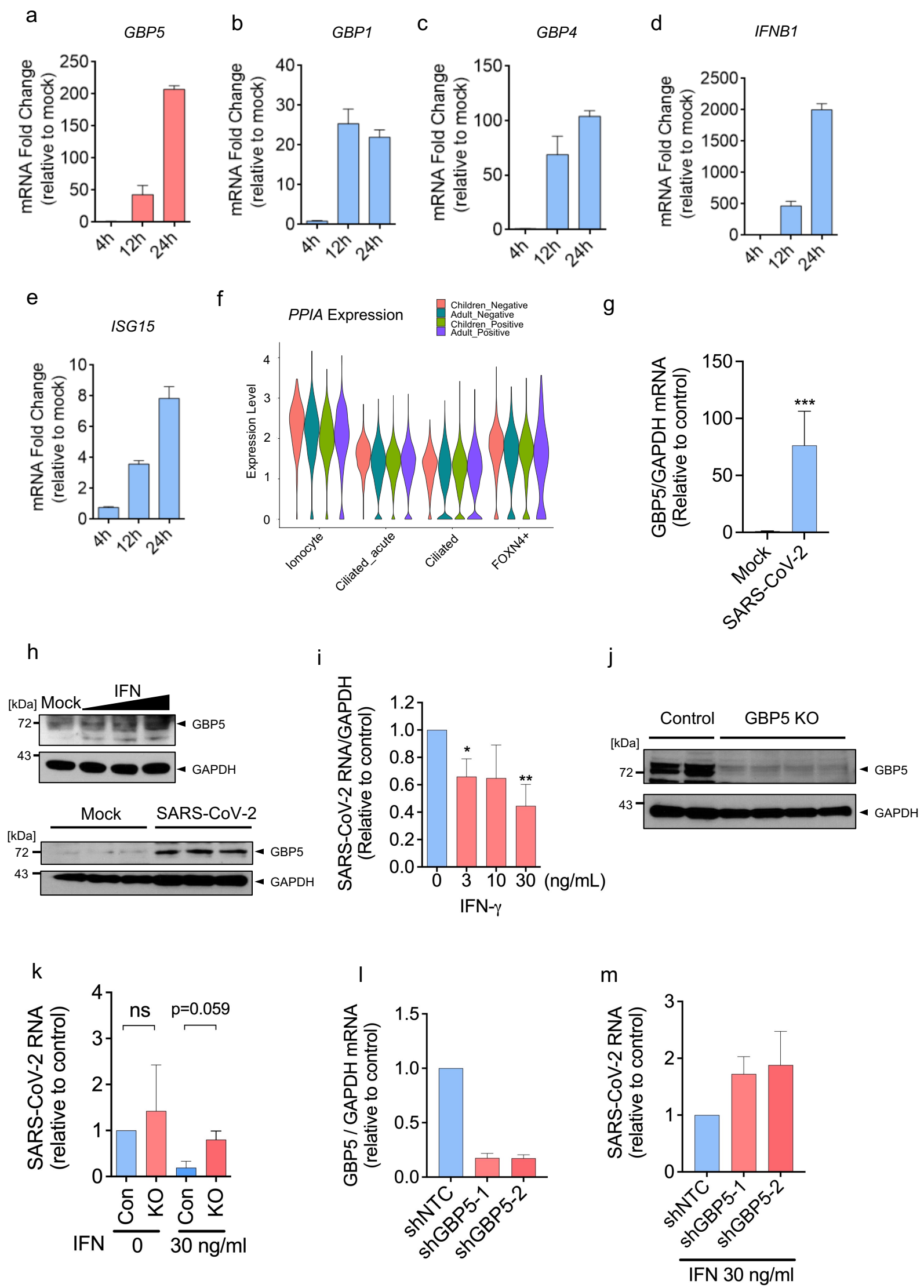

Figure S2

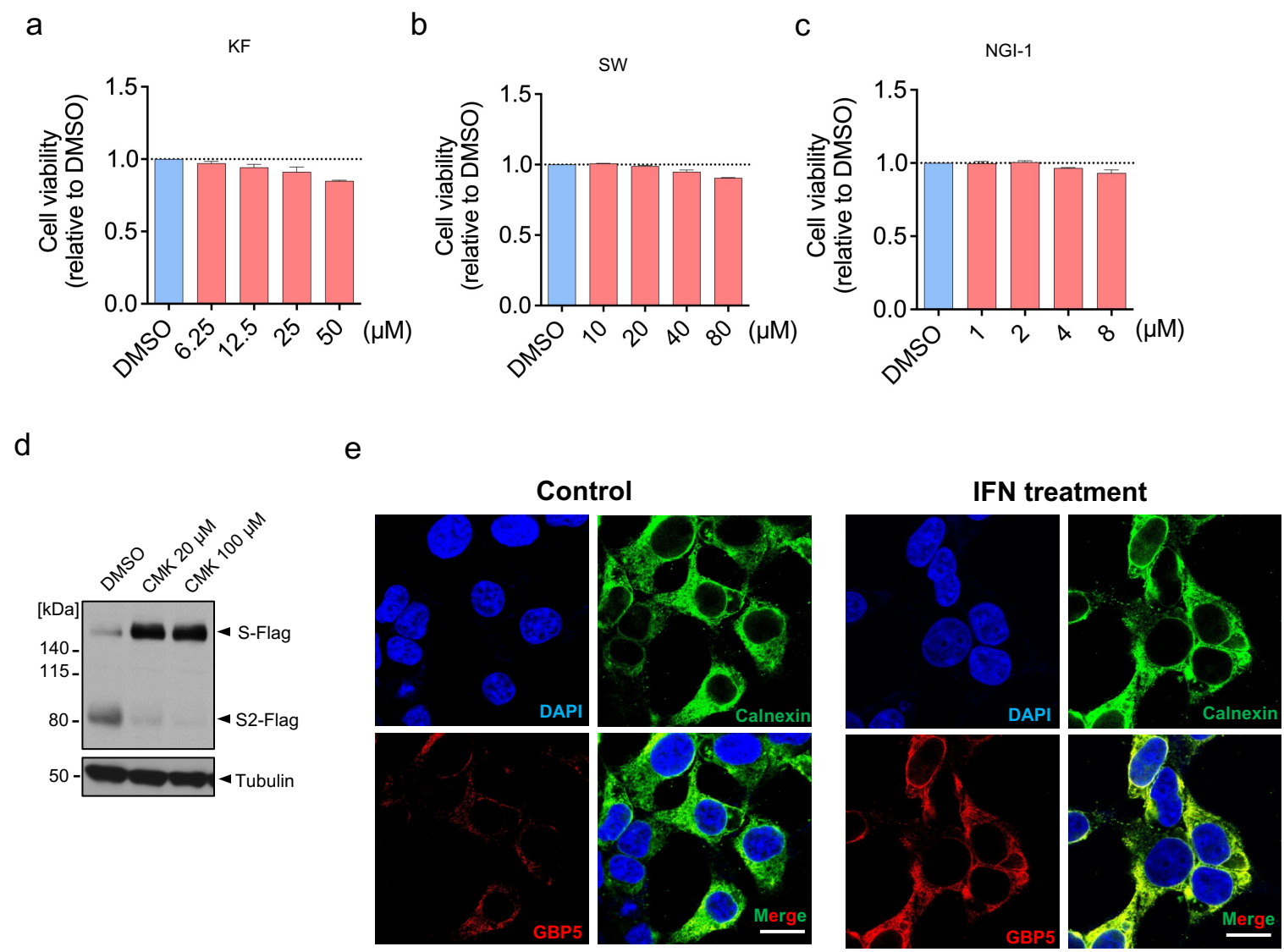

Figure S3

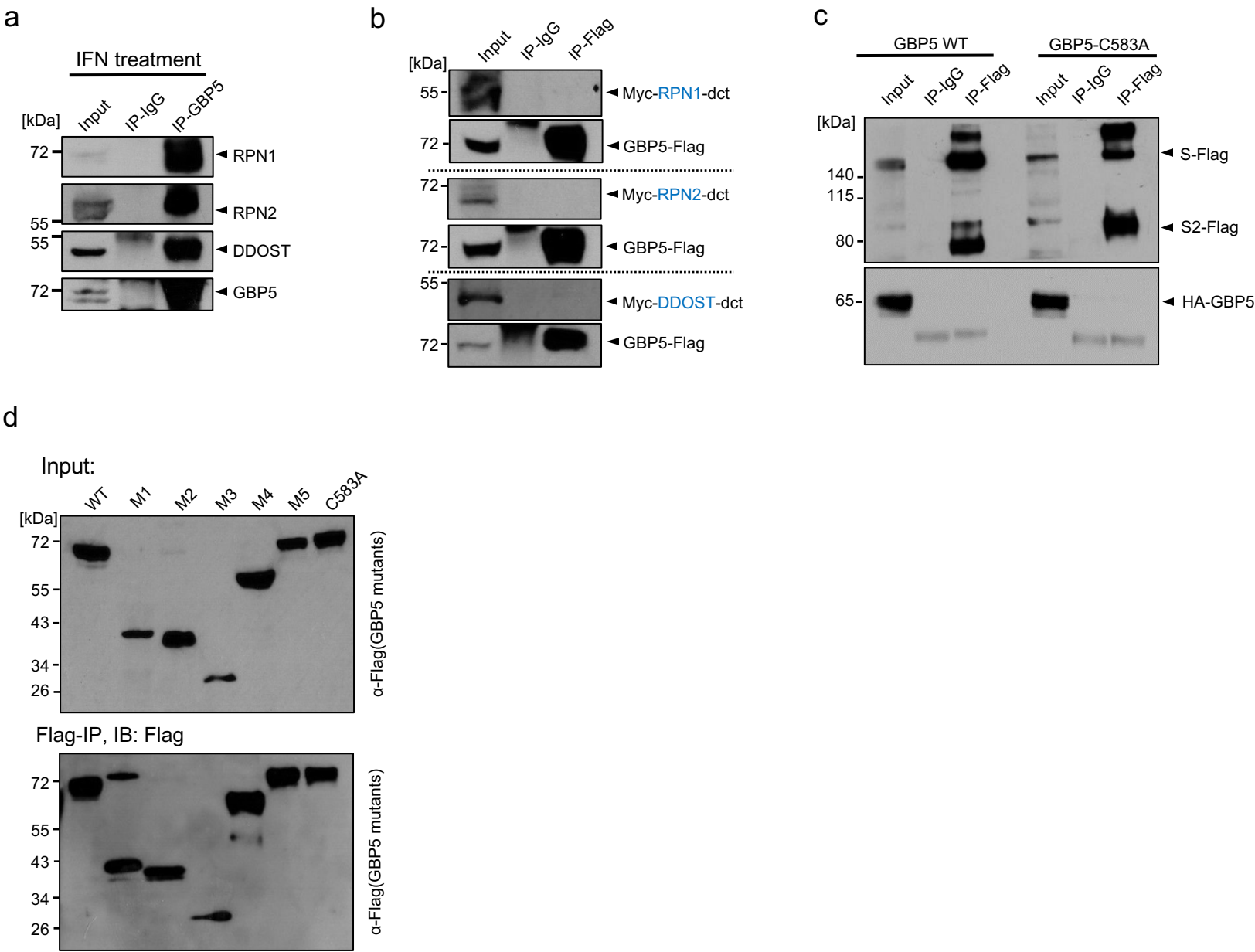

Figure S4

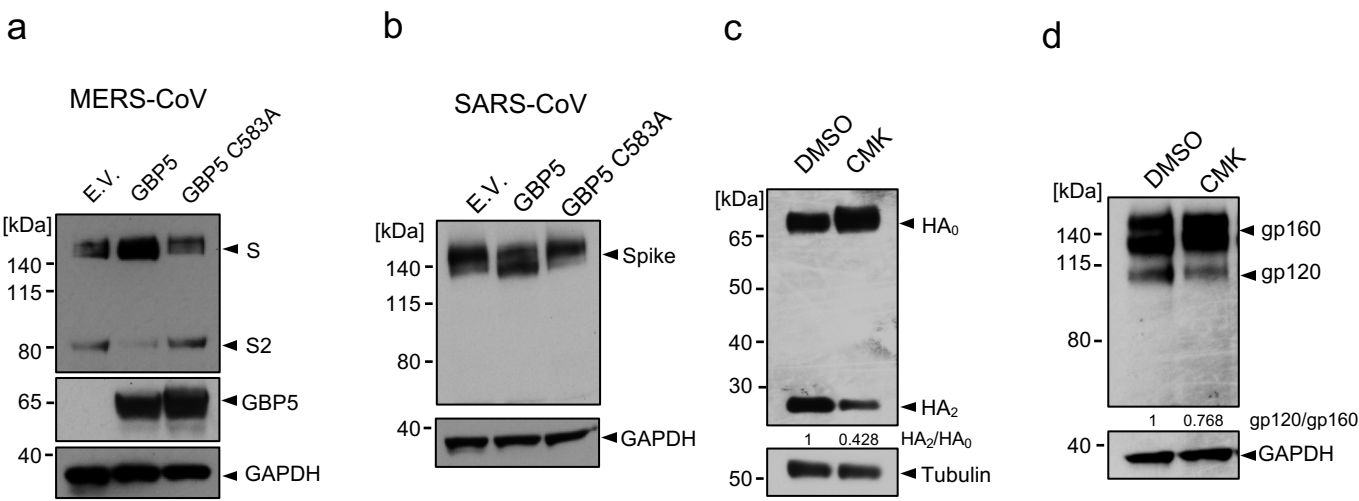

Figure S5

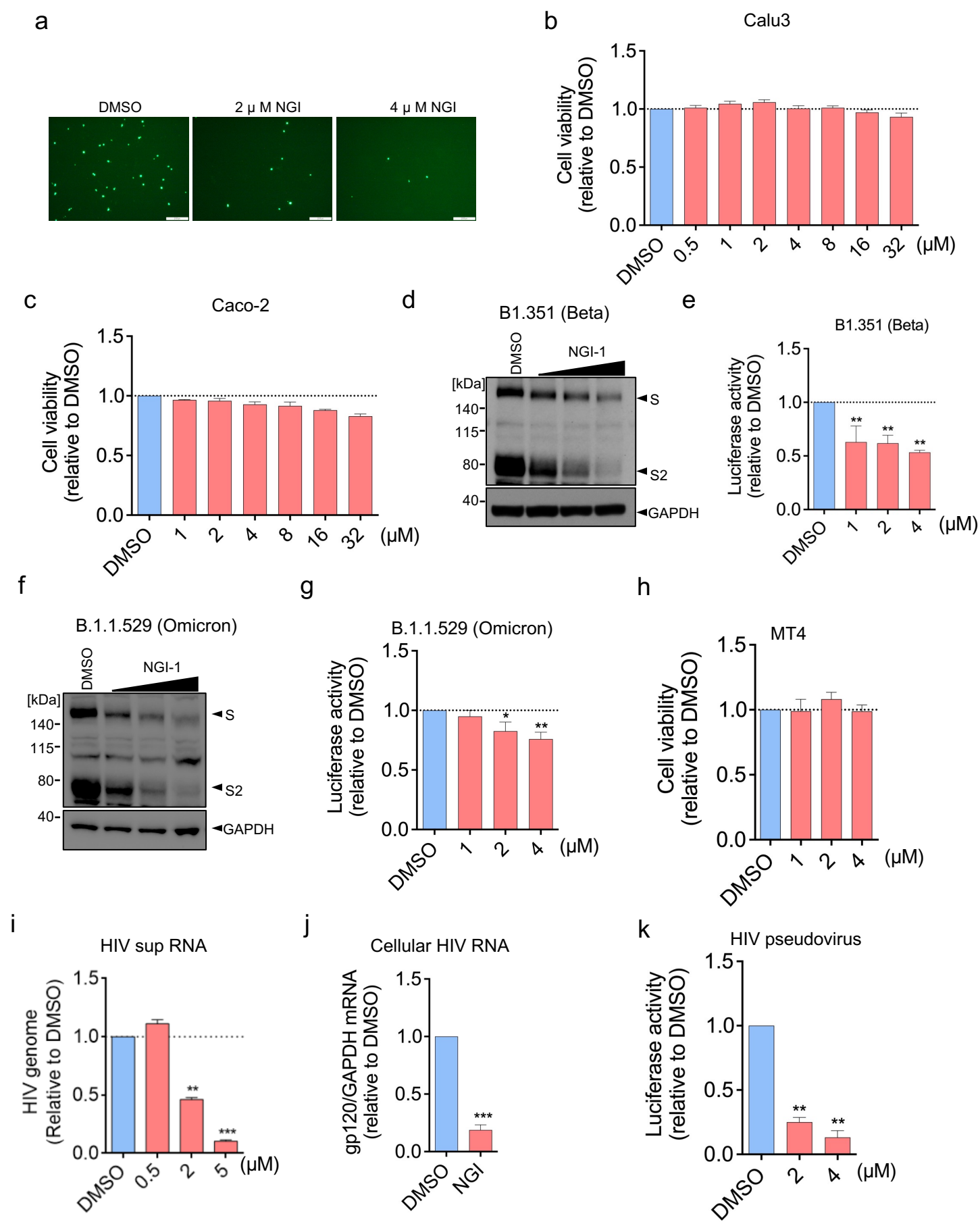

Supplement: Supplement 1 — Figure S1. GBP5 is induced by SARS-CoV-2 infection of lung epithelial cells. a–e. RT-qPCR analysis of GBP5 (a), GBP1 (b), GBP4 (c), IFNB1 (d), and ISG15 (e) mRNA levels in Calu-3 cells infected for 4, 12, or 24 h with SARS-CoV-2 WT at an MOI of 0.33. Mean ± SD of n = 2. Fold change in expression is relative to mock-infected cells. Data are from reference 59. f. Expression of the internal control gene PPIA in nasal samples from healthy or SARS-CoV-2 infected children and adults (see Figure 1 for details). g-h RT-qCPR (g) and western blot analysis (h) of GBP5 induction by SARS-CoV-2 infection or IFN-γ treatment. i. Calu3 cells were pretreated with different concentrations of IFN-γ for 16 h and infected with SARS-CoV-2 USA-WA1/2020 strain at a MOI of 0.1. After 48h, cells were collected and viral RNA in cell extracts was quantified by RT-qPCR. Mean ± SD of n = 3. *p < 0.05, **p < 0.01 by Student’s t-test. j. GBP5 KO or control Calu3 cells were treated with IFN-γ for 16 h, and the knockout efficiency of GBP5 was validated by western blot analysis. k. The viral RNA in the supernatant (see Figure 1 m–n for details) was quantified by RT-qPCR. Mean ± SD of n = 3. l-m GBP5 or control shRNA transduced human primary bronchial epithelial cells (NHBE) were infected with USA-WA1/2020 strain at a MOI of 0.1. The knockdown efficiency of GBP5 was validated by qPCR (l). The viral RNA in the supernatant was quantified by RT-qPCR (m). Mean ± SD of n = 3. Figure S2. Cytotoxicity evaluation of glycosylation inhibitors and inhibition of SARS-CoV-2 S protein cleavage by the furin inhibitor CMK. a–c. CellTiter-Glo 2.0 cell viability assay of 293T cells incubated for 24 h with DMSO or the indicated concentrations of the glycosylation inhibitors kifunensine (a), swainsonine (b), and NGI-1 (c). Mean ± SD of n = 2. d. Western blot analysis of 293T cells transfected with SARS-CoV-2 S plasmid and incubated with DMSO or CMK for 48 h. Lysates were immunoprecipitated with anti-FLAG antibody and blot [file media-1.pdf]
